# Pairmate-dependent pup retrieval as parental behavior in male mice

**DOI:** 10.3389/fnins.2014.00186

**Published:** 2014-07-11

**Authors:** Mingkun Liang, Jing Zhong, Hong-Xiang Liu, Olga Lopatina, Ryusuke Nakada, Agnes-Mikiko Yamauchi, Haruhiro Higashida

**Affiliations:** ^1^Department of Basic Research on Social Recognition and Memory, Research Center for Child Mental Development, Kanazawa UniversityKanazawa, Japan; ^2^Department of Biophysical Genetics, Graduate School of Medical Sciences, Kanazawa UniversityKanazawa, Japan

**Keywords:** parental behavior, paternal care, pup retrieval behavior, paternity, mouse

## Abstract

Appropriate parental care by fathers can greatly facilitate healthy human family life. However, much less is known about paternal behavior in animals compared to those regarding maternal behavior. Previously, we reported that male ICR strain laboratory mice, although not spontaneously parental, can be induced to display maternal-like parental care (pup retrieval) when separated from their pups by signals from the pairmate dam (Liu et al., 2013). This parental behavior by the ICR sires, which are not genetically biparental, is novel and has been designated as pairmate-dependent paternal behavior. However, the factors critical for this paternal behavior are unclear. Here, we report that the pairmate-dependent paternal retrieval behavior is observed especially in the ICR strain and not in C57BL/6 or BALB/c mice. An ICR sire displays retrieval behavior only toward his biological pups. A sire co-housed with an unrelated non-pairing dam in a new environment, under which 38-kHz ultrasonic vocalizations are not detected, does not show parenting behavior. It is important for sires to establish their own home territory (cage) by continuous housing and testing to display retrieval behavior. These results indicated that the ICR sires display distinct paternity, including father-child social interaction, and shed light on parental behavior, although further analyses of paternal care at the neuroendocrinological and neurocircuitry levels are required.

## Introduction

According to Schor and others, “a stable, well-functioning family that consists of two parents and children is potentially the most secure, supportive, and nurturing environment in which children may be raised” (Schor and American Academy of Pediatrics Task Force on the Family, 2003; Fortunato and Archetti, 2010; Benbassat and Priel, 2012). Thus, the role of a father in the home is highly significant, and currently, the physical absence of the father in the home is seen as a major problem facing families worldwide (Feinberg, 2002; Fleming et al., 2002; Amato, 2005; Benbassat and Priel, 2012; Morrongiello et al., 2013; Bornovalova et al., 2014). This raises questions regarding which factors determine paternal care and how they are maintained. This may be addressed by behavioral studies and neuroendocrinological analysis of oxytocin, stress hormones, sex hormones, or epigenetic mechanisms (Ogawa et al., 1998; Pfaff et al., 1999; Nunes et al., 2001; Gammie, 2005; Jin et al., 2007; Bridges, 2008; Nishimori et al., 2008; Lee et al., 2009; Neumann, 2009; Chourbaji et al., 2011; Douglas, 2011; Morgan and Bale, 2011; Hashimoto et al., 2012; Higashida et al., 2012a,b; Parhar et al., 2012; Soga et al., 2012; Bambico et al., 2013; Salmina et al., 2013; Morrison et al., 2014).

Although a number of animal models have been used in experimental studies of parental care (Reburn and Wynne-Edwards, 1999; Carter et al., 2009; de Jong et al., 2009; McGraw and Young, 2010; Ozawa et al., 2010; Kuroda et al., 2011; Mogi et al., 2011; Saltzman and Maestripieri, 2011; Lambert et al., 2013; Tachikawa et al., 2013; Yoshida et al., 2013), given its value for genetic studies, a mouse model of paternal behavior may be especially useful (Hager and Johnstone, 2003; Jin et al., 2007; Liu et al., 2013). While some strains of the laboratory mouse *Mus musculus* become biparental (Wright and Brown, 2000; Chourbaji et al., 2011), a phenomenon called sensitization (Rosenblatt, 1967; Rosenblatt et al., 1996), little information is available regarding the factors that specifically induce male parental behavior (Gubernick and Alberts, 1987, 1989; Lonstein and De Vries, 2000; Kentner et al., 2010; Leuner et al., 2010).

Previously, we reported that the outbred ICR strain is uniparental and is a good model for studies of parental behavior (Jin et al., 2007; Liu et al., 2008, 2013; Higashida et al., 2012a), because these mice actively reproduce offspring and exhibit easily monitored pup retrieval after separation (Fujimoto et al., 2013; Liu et al., 2013), which is a reliable indicator of parental behavior (Gammie, 2005; Wynne-Edwards and Timonin, 2007; Yoshida et al., 2013). We demonstrated that male ICR mice display robust parental care, which is induced by signaling from the pairmate dam, after separation from the pups (Liu et al., 2013). We demonstrated that this signaling is mediated through as yet unidentified olfactory pheromonal cues and auditory 38-kHz ultrasonic vocalization (USV) cues (Liu et al., 2013), that the male response can be modified hormonally via oxytocin (Akther et al., 2013), that CD38 in the nucleus accumbens is critical (Akther et al., 2013), and that the central cholinergic system is involved (Fujimoto et al., 2013). However, the factors influencing singly isolated sires in which there is no direct communicative interaction between dams and sires remain unclear.

In the present study, to simplify fatherhood evaluation, we used an all-or-nothing type of pup retrieval behavior by calculating the percentage of sires that displayed retrieval behavior (Liu et al., 2013). We investigated paternal behavior in terms of the types of conditions that can induce or maintain paternal retrieval behavior by sires when the males are isolated before the offspring are delivered by pregnant mates, and the males are held separately to prevent them being sires by physically separating them from other family members for 3 days. Then, family ties are formed with or without mate information. In other experiments, we examined isolation from pups under different housing conditions in which either pairmate dam and pup olfactory information is present or excluded.

## Materials and methods

### Animals

Male and female Slc:ICR, C57BL/6, and BALB/c mice were obtained from Japan SLC, Inc. (Hamamatsu, Japan) via a local distributor (Sankyo Laboratory Service Corporation, Toyama, Japan). The ICR mice were originally obtained from Charles River Laboratories in 1965 and since then bred in Japan with the alternative name Swiss CD1. The offspring of these mice were born in our laboratory colony, weaned at 21–28 days of age, and housed in same-sex groups of 3–5 animals until pairing (Liu et al., 2013). The animals were paired and kept in our laboratory under standard conditions (24°C; 12-h light/dark cycle, lights on at 08:00) with food and water *ad libitum*. The mice were housed together continuously in standard mouse maternity cages. The experiments were performed in accordance with the Guidelines for the Care and Use of Laboratory Animals of Kanazawa University.

### Behavioral testing

Virgin males and females were paired at 45–55 d. A single male and a single female were continuously housed together in a standard mouse maternity cage from the mating period until the delivery of pups. In some experiments, the males were separated in new cages 1 day before parturition to prevent formation of family relationships and kept in the new cages for 3 days. Then, the males were allowed to meet their pups with or without pairmates from day 3 to day 5. All family units composed of a new sire (first-time father), dam, and their first litter were experimentally naïve.

One male parent was placed for 10 min in the original cage or new cage alone or with his pairmate (separation environment). Five pups were randomly selected from the litter and placed individually at a site remote from the nest in the original cage. The sires were returned to the original home cage or a new cage in the presence of their five biological or foster pups to assess parental behavior. Parental retrieval behavior (percentage of sires exhibiting retrieval) was examined for 10 min following reunion. The behavioral tests were performed in a randomly mixed sequence of experimental groups. Experiments were usually performed at 10:00–15:00. We defined retrieval as positive if the sires carried all 5 pups to the original nesting place or within two thirds of the distance between the nest and the place at which the pups had been placed (Liu et al., 2013). We also observed other parental behaviors (grooming, crouching, and huddling) as defined by Gubernick and Alberts (1987, 1989). The animals in this and subsequent experiments were tested only once.

### Measurement of USVs

Experiments were carried out in a soundproof chamber measuring 600 × 500 × 500 mm (model MC-050/VA; Muromachi Kikai, Tokyo, Japan). USVs were detected with a condenser microphone (Type 7016; Aco, Tokyo, Japan) and a preamplifier (type 4116; Aco) designed for sound pressure level (SPL) measurements between 20 Hz and 90 kHz. A 4-kHz band-pass filter was used to minimize background noise during recordings; however, most WAV files still contained a considerable amount of “non-USV” signal. Extraneous noise was identified and removed from the sonograms as far as possible. When a rater found an ultrasound signal that was difficult to interpret, the call was evaluated by a minimum of one additional trained observer and identification required a consensus by all raters. Each sonogram was then evaluated with a series of automated parameters. The microphone was placed 50 cm above the cage in a soundproof chamber and connected to an amplifier (model UMA-2; Muromachi Kikai). Acoustic signals were transmitted to a vocalization analyzer system (model MK-1500; Muromachi Kikai) with functions such as an analog-to-digital converter (192 kHz), frequency filters, a digital fast-Fourier-transform analyzer, and signal input—output terminals. Input signals were visualized on SpectraLAB (Sound Technology Inc., State College, PA) in the analyzer system on a personal computer. USVs were recorded as WAVE files and analyzed; the number of calls, frequency, and wave width (>40 ms) were measured using a USV monitor (Muromachi Kikai).

### Statistical analysis

The data were calculated as the means or the means ± s.e.m. Two-tailed Fisher's exact probability test was used for single comparisons of retrieval behaviors. The remaining data were analyzed by two-tailed Student *t*-test.

## Results

It has been reported that parental behavior in mice is dependent on the strain (Wright and Brown, 2000). Therefore, we first examined and compared parent–pup family units in three strains, i.e., ICR, C57BL/6, and BALB/c mice, under various experimental settings. The data are summarized in Table 1. Maternal nurturing behavior was observed in dams of all three strains, in a strain-nonspecific fashion, except for the low rate of retrieval by the BALB/c dams. In contrast, paternal behavior was variable between the strains. No retrieval behavior was observed by BALB/c sires (*n* = 15). C57BL/6 sires displayed retrieval during reunion after single-separation in new cages (approximately 40%, *n* = 15). However, isolation together with the partner in new cages did not potentiate but rather decreased this rate to 13.3% (*n* = 15). This parental behavior suggests that C57BL/6 males display mate-independent paternal behavior. Interestingly, 38-kHz USVs were not recorded from any dam–sire pairs of C57BL/6 and BALB/c strains separated in new cages for 10 min. These results indicated that pairmate-dependent care is specific to the ICR strain. Therefore, in the following experiments, we examined various critical conditions under which ICR strain males did or did not show paternal behavior.

**Table 1:** **Parental behaviors in three strains of mice**.

**Behavior**		**Mouse strain**		
		**ICR (***n*** = **15**)**	**C57BL/6 (***n*** = **15**)**	**BALB/c (***n*** = **15**)**
Dam	Retrieval	100% fast, rhythmic	100% fast, rhythmic	60% slow, interrupted
	Crouching	Over all pups	Over not all pups	Over all pups
	Grooming	Rare	Rare	Rare
	Nest building	Sometimes	Sometimes	Sometimes
Sire	Retrieval by separation	10%	40%	0%
	After co-housing pairmates	60%	10%	0%
		Fast (<4 min)	Very slow	–
		Smooth	Intermittent	–
	Crouching	Over not all pups	Not often	–
	Grooming	Rare	Rare	–
	Nest building	Not often	Rare	–
Pup	Number of pups per litter	~15	~5	~7
	Survival ratio	~100%	60–70%	~100%
	USVs	>70 calls/2 min	<20 calls/2 min	>80 calls/2 min
Communication from dams to sires with 38 kHz USVs		Detected	Not detected	Not detected
Pattern of paternal care		Mate-dependent	Mate-independent	None

### Retrieval behavior by sires separated alone in home cages

**Figure 1:**
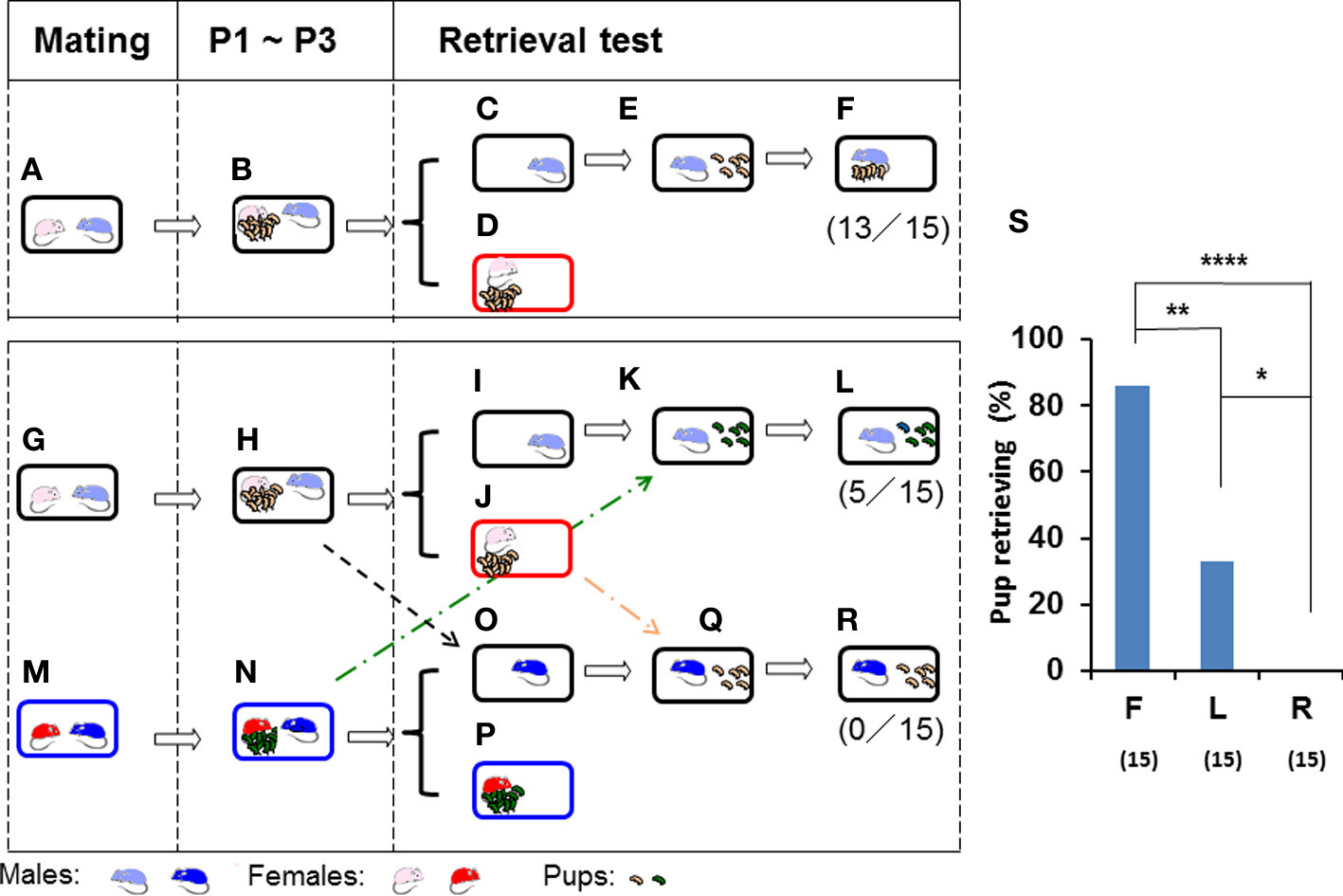
**Parental retrieval test in ICR mice for biological and non-biological pups**. Schematic representations of the parental care test in three mated pairs **(A,G,M)**. After cohabiting with their pups as a family for 3 days from postnatal day 1 (P1) until postnatal day 3 (P3) **(B,H,N)**, the sires were separated in the home cage **(C,I,O)** from the pups and pairmates **(D,J,P)** for 10 min. The sires were then reunited with five biological **(E)** or non-biological **(K)** pups. Subsequent pup retrieval behavior over a 10-min period was then observed **(F,L)**. The third sire **(M,N)** was placed in the home cage **(O)** of another family **(H)**, and retrieval was tested for non-biological (another family's) pups in an unrelated cage **(Q,R)**. The numbers of positive mice/number of mice tested are shown in parentheses. The number of sires displaying retrieval behavior out of sires tested was expressed as a percentage **(S)**. *N* = 15 for each experiment. Two-tailed Fisher's exact probability test: between sires toward biological **(F)** and non-biological **(L)** pups or unrelated sires **(R)**, ^**^P < 0.01 and ^****^P < 0.0001, respectively; and between sires tested toward non-biological pups **(L)** and unrelated sires **(R)**, ^*^P < 0.05.

The experimental paradigms for each experiment are shown schematically in each figure. In Figure 1, we first reproduced our previous results (Liu et al., 2013). Male and female ICR strain mice were paired and housed together continuously in a standard mouse maternity cage (Figure 1A). The mice were left undisturbed during the first 3 days after the birth of their pups (Figure 1B), during which they displayed distinct paternal and maternal behaviors as described previously (Liu et al., 2013). The sire and dam nursed the pups. This involved nest-building, pup retrieval, licking, and huddling over the pups and lactating. However, as described in the Methods section, we mainly analyzed the male's retrieval behavior, as a parental role, in the following experiments.

The sire in the first family was left alone in the vacated cage during the period of separation (Figure 1C), whereas the pups and dam were removed and placed in a new cage (Figure 1D) separated from the family cage. After 10 min, the five selected pups of the sire (biological offspring) were returned to the nursing cage in a remote area away from the nest, where the sire was present (Figure 1E). The sire retrieved the offspring over 10 min (86% of the sires, *n* = 15; Figures 1F,S).

If the non-biological (foster) pups (Figure 1N) of the third family (Figures 1M,N) were introduced into the vacated home cage with the second sire (Figures 1I,K) in the second family (Figures 1G,H), instead of the biological pups (Figures 1H,J), 33% of the 15 sires displayed pup retrieval (Figures 1L,S; two-tailed Fisher's exact probability test between sires toward biological (F) and non-biological (L) pups, *P* < 0.01).

When a sire from the third family (Figure 1N) was placed and isolated for 10 min in the home cage of the second family (Figure 1O), the third sire did not retrieve any of the foster (second family's) pups (Figures 1R,S; *n* = 15, two-tailed Fisher's exact probability test between unrelated sires (R) and sires with non-biological (L) or biological (F) pups, *P* < 0.05 and *P* < 0.0001, respectively). These results suggested that paternal pup retrieval behavior in the home cage is maintained by biological family cues of their mate dams and remaining pups.

### Retrieval behavior by sires after separation in new cages

**Figure 2:**
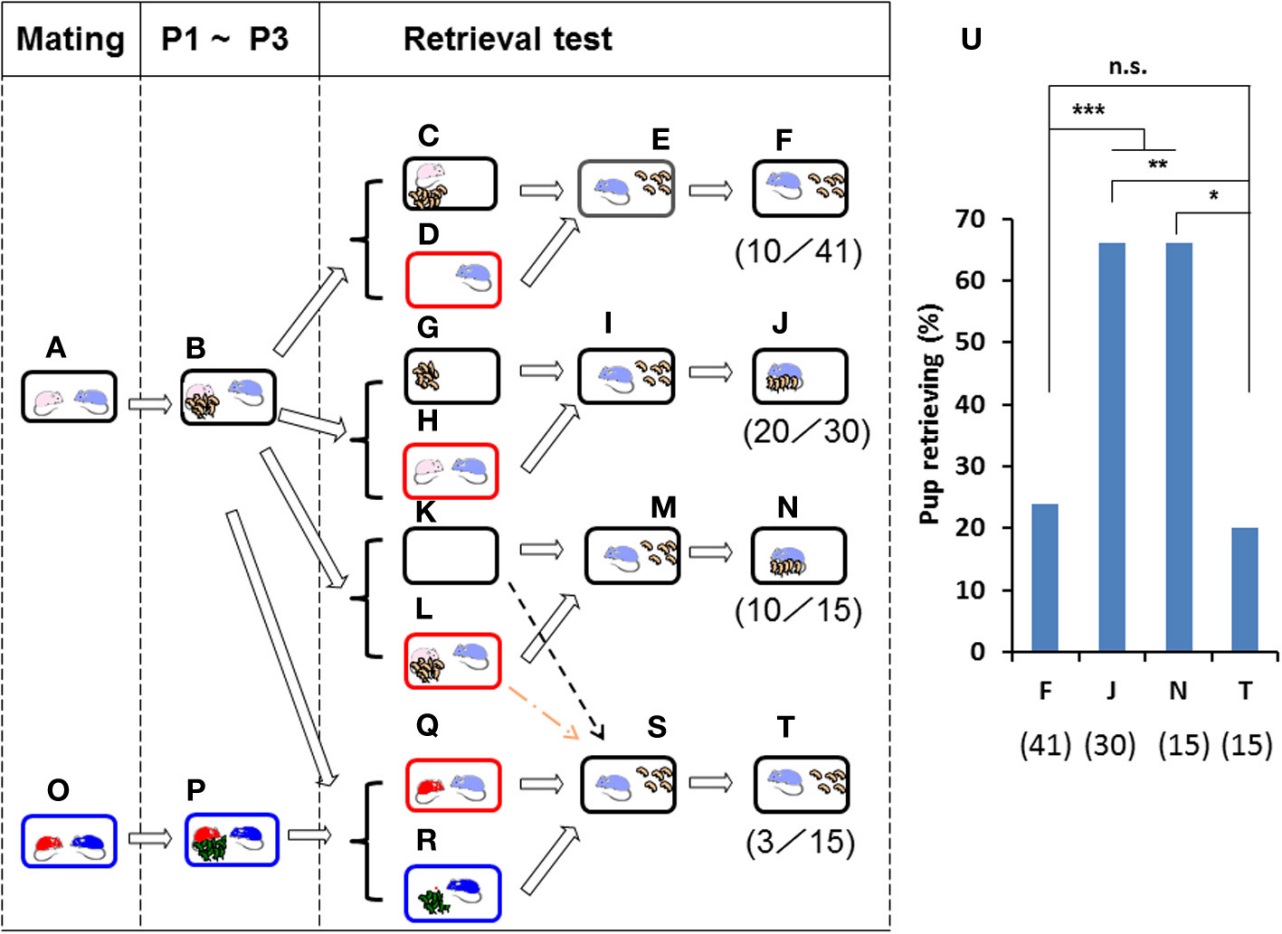
**Paternal retrieval test in ICR mice isolated in new cages**. Paired couples were kept in rearing cages from mating to postnatal day 3 (P3) **(A,B,O,P)**. In **(C,D)**, the pups and the mating dam were left in their home cages **(C)**, and the sire alone was placed in a new holding cage **(D)**. In **(G,H)**, the pups were kept in the original cage, and the parents were placed in a new cage **(H)**. In **(K,L)**, the whole family was moved to a new cage **(L)**. In **(Q,R)**, the sire was kept during the separation period **(Q)** with a non-mating dam of another family **(O,P)**. After isolation for 10 min in each cage, pup retrieval behavior over a 10-min period was observed in each case **(E,I,M,S)**. The number of sires displaying retrieval behavior was scored **(F,J,N,T)**. The numbers of positive mice/number of mice tested are shown in parentheses and expressed as percentages **(U)**. Two-tailed Fisher's exact probability test: between sires separated alone **(F)** and together **(J)** or as a whole family **(N)**, ^***^P < 0.001 equally; between sires separated together **(J)** and as a whole family **(N)**, ^**^P < 0.01; between sires separated alone **(F)** and co-housed with unrelated dams **(T)**, not significant (n.s.); sires separated as a whole family **(N)** and co-housed with unrelated dams **(T)**, ^*^P < 0.05.

Male parental care in Figure 1 may have been induced by the fact that the males were left in the nursing environment during parent–pup separation. To select out pup information during isolation, we used the co-housing paradigm presented in Figure 2. We examined whether sires developed paternal behavior following time spent with the family. Pup retrieval increased on a daily basis after parturition, while dams displayed a higher retrieval ratio from the first day of parturition than the sires (Table 2).

**Table 2:** **Percentages of sire's or dam's exhibiting retrieval behavior during the postpartum period**.

**Postnatal day of pups**	**Percentage of exhibiting retrieval behavior**	
	**By sires**	**By dams**
1	14 (15)	55 (20)
2	40 (20)	90^*^ (20)
3	65^**^ (20)	90^*^ (20)
4	70^**^ (17)	85 (20)
5	65^**^ (20)	75 (16)

Number of mice tested are shown in parentheses. ^*^, ^**^Significantly different from day 1,

* P < 0.05 and

** P < 0.01, respectively, two-tailed Fisher's exact probability test.

The sires alone (Figure 2D) or together with the mate dams (Figure 2H) were placed in a new cage for 10 min, whereas the pups alone (Figure 2G) or together with dams (Figure 2C) were left in the home cage. Then, the sires were returned to the home cages in which five pups remained (Figures 2E,I). The male's retrieval behavior was undiminished when housed with the pairmate (66%, *n* = 30; Figures 2J,U) but was strongly reduced when housed alone (24%, *n* = 41; Figures 2F,U). As expected, a high level of sire care was displayed after isolation in the new environment together with mate dams and pups (as the whole family (Figures 2K,L) (66%, *n* = 15; Figure 2U): two-tailed Fisher's exact probability test between sires separated alone (F) and together (J) or as a whole family (N), *P* < 0.001, equally.

The latter was specifically associated with co-habitation with the pairmate dam during the separation period (Figure 2H), because negligible retrieval behavior was apparent if the sire was housed with the dam of another brood (Figures 2O–T; 20%, *n* = 15); two-tailed Fisher's exact probability test shows no significant difference between sires separated together with unrelated dams (T) and alone (F); and separated together (J), *P* < 0.01; and separated as a whole family (N), *P* < 0.05, Figure 2U). Thus, it appears that the mate dam provides some signal(s) during the separation period to induce parental behavior in the sire, in agreement with the results reported previously (Liu et al., 2013). Whereas parental care by the dam is independent of the presence of the male or the housing environment, that by the male is strongly dependent on cues from the pairmate dam and/or home cage.

We recorded USVs (with >40 ms in wave width) to determine their role as one form of critical interactive information in this paradigm. We detected 38-kHz USVs identical to those reported previously (Liu et al., 2013) under isolation conditions in new cages for 10 min between sires and mate dams at a frequency of 25.9 ± 4.8 calls/10 min (*n* = 8, Table 3; *P* < 0.01 from other values, two-tailed Student *t*-test). No identical 38-kHz USVs were recorded between sires and unrelated dams. Instead, 30–80-kHz USVs were recorded infrequently at 40.7 ± 26.7 calls/10 min (*n* = 11) between unfamiliar couples. These 30–80-kHz USVs were emitted when a sire was co-housed with a virgin female at 313.6 ± 64.9 calls/10 min (*n* = 11, *P* < 0.001 from two other values, two-tailed Student *t*-test). These data clearly support the suggestion that paternal retrieval is essentially triggered by the pairmate's 38-kHz USVs.

**Table 3:** **Number of USVs recorded from cages of sires co-housed with different types of females for 10 min**.

	Type^*^	**Number of USVs (calls/10 min)**	**(*n*)**
	**38-kHz**	**30–80-kHz**	
With pairmate dam	25.9 ± 4.8^**^	0	(8)
With unrelated dam	0	40.7 ± 26.7	(11)
With virgin female	0	313.6 ± 64.9^***^	(11)

USVs (with >40 ms in duration) were recorded in n pairs.

* Judging from the previous results (Liu et al., 2013), 38-kHz USVs appear to be emitted from pairmate dams and 30–80-kHz USVs from sires.

** P < 0.01 or

*** P = 0.001, from pairmate dams, unrelated dams or virgin females, respectively, two-tailed Student's t-test.

### Retrieval by isolated before pairmate parturition

**Figure 3:**
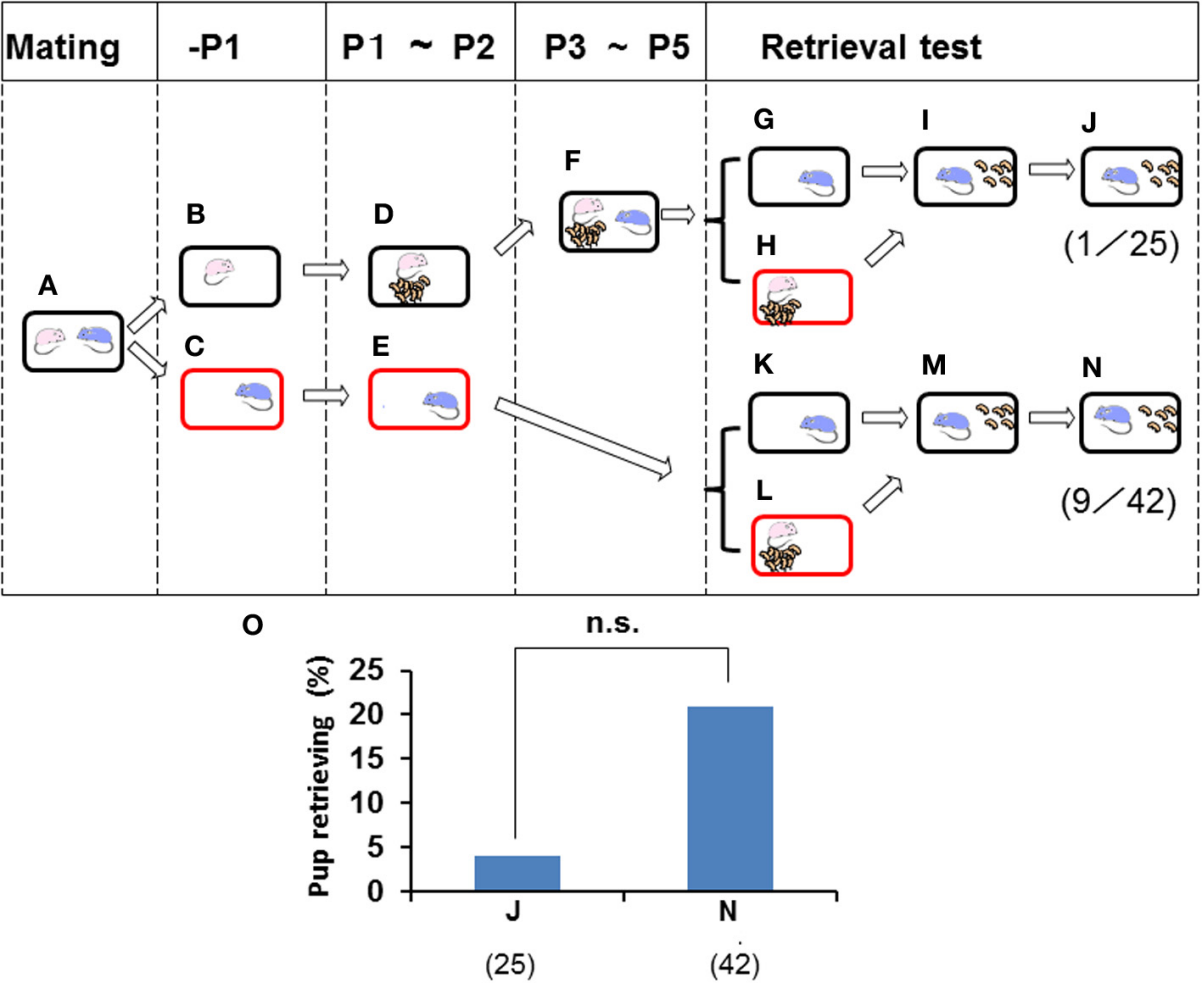
**Paternal retrieval test in ICR mice isolated prior parturition from the mating pair and then united as a whole family**. A paired couple was kept in a rearing cage from mating **(A)** to 1 day before parturition, and the female and male were then kept in a home cage **(B)** or in a new cage **(C)**. The next day, the female delivered her pups **(D)** and remained until postnatal day 2 (P2). The male was kept continuously in the new cage until P2 **(E)**. From P3 to P5, the sire was introduced to the family cage with the dam and pups **(F)**. In another experiment, pup retrieval behavior over a 10-min period was examined for sires at P2 **(K–N)** or at P5 **(G–J)**. The number of sires displaying retrieval behavior was scored **(J,N)**. The numbers of positive mice/number of mice tested are shown in parentheses and expressed as percentages **(O)**. Note that two values in O are equally very low: no significance (n.s.) between **(J)** and **(N)**, two-tailed Fisher's exact probability test.

**Figure 4:**
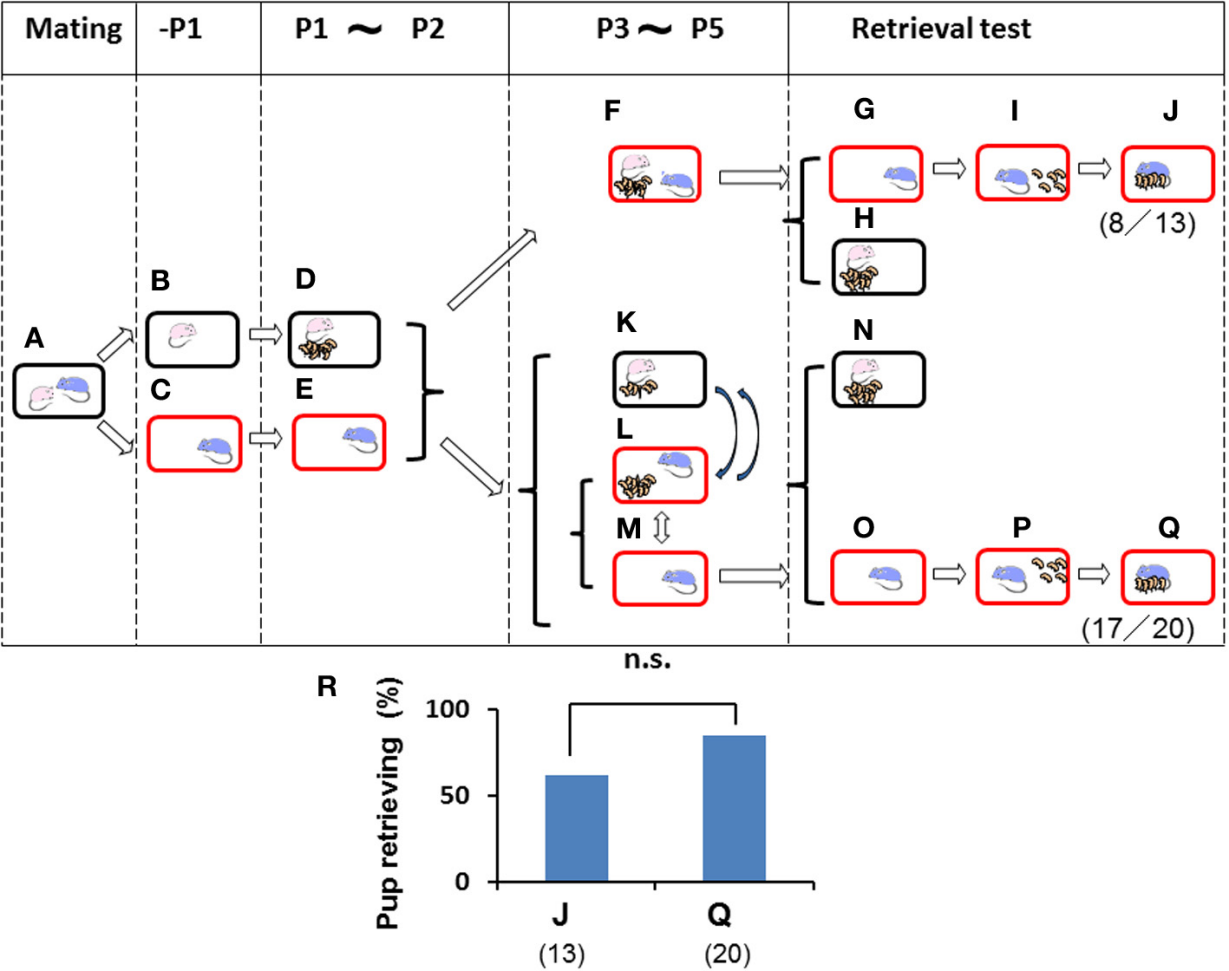
**Paternal retrieval test in mice isolated prior parturition from the mating pair and then united as a whole family or with pups only**. A paired couple was kept in a rearing cage from mating **(A)** to 1 day before parturition. The female was kept in a home cage **(B)** and delivered her pups **(D)** and remained until postnatal day 2 (P2) **(D)**. The male was kept in a new cage before meeting the pups **(C)** and kept until P2 **(E)**. The dam and pups were introduced in the sire's own (new) cage and stayed as a whole family until P5 **(F)**. Instead of the whole family, in another experiment, only pups in home cages with their dams **(K)** were transferred twice for 3 h (total 6 h) a day to the sire's cage **(L)**. During the rest of the time from P3 to P5, the sire stayed alone **(M)**, and pups were located with the dam **(K)**. Pup retrieval behavior over a period of 10 min was examined (**G–J** and **N–Q**, respectively). The number of sires displaying retrieval behavior was scored **(J,Q)**. The numbers of positive mice/number of mice tested are shown in parentheses, and the numbers of sires displaying retrieval were expressed as percentages **(R)**. Note that the retrieval rate in two cases **(J,Q)** was high enough to have no significance (n.s.), two-tailed Fisher's exact probability test.

The retrieval behavior displayed by males may have been induced by family formation in the nursing cage environment. To assess this possibility, data were obtained from parting males (Figures 3, 4) that remained with the paired pregnant females 1 day before parturition of their first litter and were then separated into a new cage (Figures 3A–C). The males were then isolated alone for 3 days (Figure 3E). When sire paternal retrieval was examined immediately on day 3 in the sire home cage (Figure 3K), 21.4% of sires with no prior contact with their biological pups and pairmate dam, i.e., the paternity unformed state (Figure 3E), displayed retrieval behavior (*n* = 42; Figure 3N). Next, when the isolated males were relocated in the home cage and stayed with the family (pups and pairmate dam) for 3 days (Figure 3F), the rate of retrieval in their home cage was only 4% (*n* = 25; Figures 3G–J). Although the sire lived together with the family for 3 days, such treatment made no contribution to the formation of paternity (two values in Figure 3O were equally very low; no significance, two-tailed Fisher's exact probability test).

To further analyze the relevance of family interaction during the stay as a whole family on postnatal days 3–5 (P3–P5) (Figure 3), we used the short-term pup exposure method (twice for 3 h for a total 6 h a day; Figure 4) to acquire or learn the process of paternity for the family. Males were isolated in new cages prior to parturition (Figures 4B,C) and kept in the cages for 2 days (Figures 4D,E). Then, pairmate dams and pups were relocated to the male's cage, and the whole family was kept there for 3 days (Figure 4F). Retrieval behavior was displayed by 8 (62%) of 13 sires (Figures 4G–J). The high level of retrieval appears to have been caused by continuously living in new cages that had been established as the male's territory.

In this suitable condition, we examined whether the presence of the dam was necessary for parental behavior by the isolated males. From P3 to P5, the pups and dam were kept together in their original home cages (Figure 4K), but the pups were temporarily transferred to the sire's cage twice for 3 h (a total of 6 h) per day (Figure 4L), and the males were otherwise alone for the rest of the day (18 h; Figure 4M). These sires showed retrieval behavior at a very high rate (17 (85%) of 20 sires tested; Figures 4O–Q). In both cases, the sires displayed a very high frequency of retrieval after living as the whole family or only with pups shortly in new cages that had, nevertheless, been established as the territory and established nest of the male, although no significant differences were observed between two types of sire (J and Q in Figure 4R; not significant, two-tailed Fisher's exact probability test). Furthermore, these results indicated that direct interaction with the mate dam is not necessary if the home territory is established by the sires.

**Figure 5:**
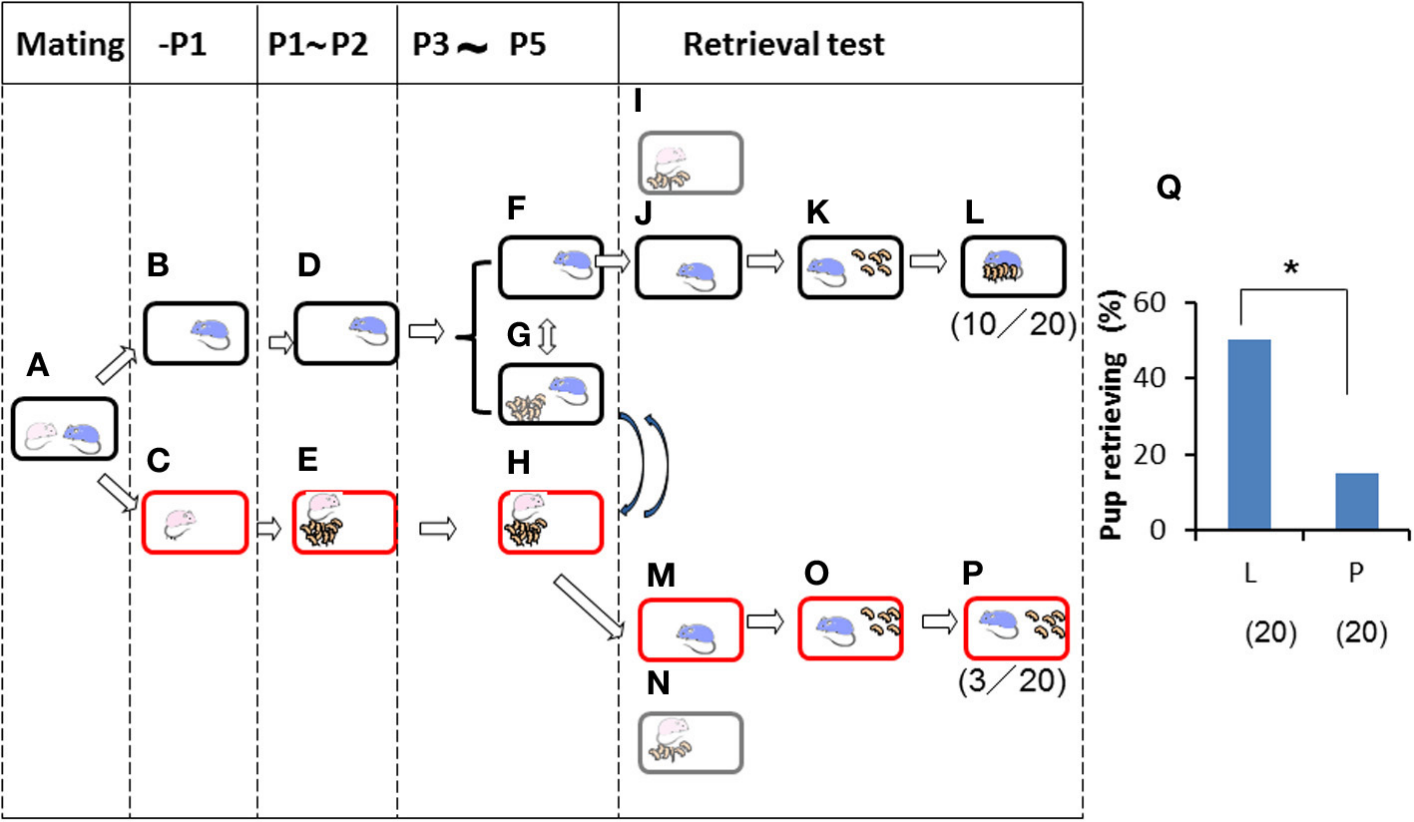
**Paternal retrieval test in ICR mice isolated prior parturition from the mating pair and then united with pups only**. A paired couple was kept in a rearing cage from mating to 1 day before parturition **(A)**. The male was kept in the old cage **(B)** before meeting the pups and kept until P2 **(D)**. The female was kept in a new cage **(C)**, delivered her pups, and remained until postnatal day 2 **(E)**. The pups were transferred twice for 3 h (total 6 h) a day **(G)** from the dam's (new) cage **(H)** during P3 to P5. During the rest of the time, the sires stayed alone in the home cages **(F)** and the dams were with the pups **(H)**. Pup retrieval behavior over a period of 10 min was examined at P5 (**I–L** and **M–P**, respectively). The number of sires displaying retrieval behavior was scored **(L,P)**, and the numbers of positive mice/number of mice tested are shown in parentheses. Pup retrieval was expressed as percentages (**Q**; ^*^P < 0.05 between old **(L)** and new **(P)** cages, two-tailed Fisher's exact probability test).

Finally, we further examined the impact of territorial information on male retrieval behavior. Family cues were learned by individual sires in a manner identical to that shown in Figure 4 (Figures 5A–F) during P3–P5, but in this case, via short exposure by transferring of their biological pups with their dams in new cages to the nursing cage with the sires. Then, retrieval behavior was examined under two housing conditions: in the sire's home cage in which the sire had stayed continuously (Figures 5F,G,I–L), or in a new cage (to the sires) in which the mate dams and pups had been staying (Figures 5G,H,M–P). In the home cages, 10 (50%) of 20 sires showed retrieval (Figure 5L), whereas only 3 (15%) of 20 sires in new cages displayed retrieval behavior (*P* < 0.05 between testing in old (L) and new (P) cages shown in Figure 5Q, two-tailed Fisher's exact probability test). In both cages, nests were established by the sire and dam. However, the new cages established by the sires' mate dams were quite new to the sires, even if the cages were fully filled with the mate dam's olfactory information.

## Discussion

The studies described here were performed to test several hypotheses that had not been explored previously (Akther et al., 2013; Fujimoto et al., 2013; Liu et al., 2013), pertaining to the various conditions responsible for parental behaviors other than the communicative interaction between sires and dams. Four findings are of particular interest: (1) among the mouse strains tested, the mate-dependent paternal retrieval behavior was observed only in the ICR strain (Table 1), and acquisition of such paternal behavior increased slowly following parturition of the dam (Table 2); (2) the ICR sires displayed parental retrieval behavior only for their own biological pups (Figure 1); (3) interaction between the sires and unrelated non-mating dams is not effective (Figure 2) and does not involve 38-kHz USVs (Table 3); (4) it is important for the sire to establish its home cage (territory) by continuous housing to display parental retrieval behavior (Figures 3–5).

After separation from pups in the home or new cages with the sires alone or together with the pairmate dam, the sires displayed retrieval behavior, as shown in Figures 2J,N, in agreement with previous reports (Liu et al., 2013). We designated this behavior of the sire as mate-dependent parental behavior. In the present study, this particular behavior was specific to the ICR strain and was not observed in two other laboratory strains, i.e., C57BL/6 and BALB/c mice. Therefore, the ICR strain's mate-dependent retrieval is not a general behavior observed equally in all mice but is strain-specific. However, this does not reduce the value of our findings because the observed paternal behavior is unique. Furthermore, when considering human society, human males are not completely and genetically predisposed to display parental behavior. In this context, the behavior of the ICR strain may be a more suitable and novel model for investigating paternal behavior, comparing the genetically determined paternity, observed in animals such as voles or California mice (de Jong et al., 2009; Ahern et al., 2011).

The ICR sires displayed parental retrieval behavior only for their biological pups, indicating that they can discriminate between their biological and non-biological offspring. This discrimination likely depends on odor or USV (Kuroda et al., 2007, 2011). The characteristic 38-kHz USVs were not recorded during co-housing of ICR sires with non-mate dams, suggesting that the sires can distinguish the mate from non-mate dams or that the dams can distinguish the mate from non-mate sires. These results strongly support our suggestion that 38-kHz USVs are critical and have context for sires to induce retrieval behavior.

In these experiments, we examined the olfactory information of pups and cages (homes) for the sires prior to separation from the mate dam and their offspring. In habituation as a family, the presence of the mate was not completely essential. Interestingly, we estimated that the territory information is much more important to sires than the pheromones in the cages once they had established their home cage. Surprisingly, when the sires were continuously housed in their newly established home cages, they displayed paternal retrieval. In sharp contrast, if the cage was new to the sire, even though the dam's and sire's olfactory information was there, the sire failed to display retrieval behavior. These observations suggested that territory establishment is critical to maintaining paternity (Wright and Brown, 2000).

Pup retrieval as a parental behavior is rare among laboratory mice that are not genetically monogamous (Wright and Brown, 2000; Kalueff et al., 2007). We found conditions in which the ICR sires retrieved their pups related to their family structure. This unique ability of the ICR sires will contribute to the increased survival rate after reproduction and to the high level of social attachment and interaction. We have recently reported that central cholinergic cellular signaling (Fujimoto et al., 2013) and CD38 and oxytocin signaling in the nucleus accumbens (NAcc) (Akther et al., 2013) are critical for the expression of paternal care of the ICR mice. We also demonstrated the modulatory roles of the mPOA and VP on parental behavior in rodents (Akther et al., 2014). These published findings suggest that the neural circuitry mediating paternal behavior includes the mPOA, VTA, NAcc, and VP, and may be similar to those that mediate maternal behavior as proposed by Numan and others (Numan et al., 2005; Lee and Brown, 2007; Wynne-Edwards and Timonin, 2007; Numan and Stolzenberg, 2009). In addition, it is particularly interesting to test if mPOA galanin neurons regulate mate-dependent parental behavior in the ICR strain (Wu et al., 2014). Further neuroendocrinological and neurocircuitry analyses in ICR mice will be useful for understanding disorders with social impairment, such as autism spectrum disorders and schizophrenia (Insel, 2010; Munesue et al., 2010; Riebold et al., 2011; Feldman et al., 2012; Salmina et al., 2013).

## Author contributions

Haruhiro Higashida designed experiments. Mingkun Liang, Jing Zhong, Hong-Xiang Liu, Olga Lopatina, Ryusuke Nakada, Agnes-Mikiko Yamauchi, and Haruhiro Higashida performed animal experiments. Haruhiro Higashida and Mingkun Liang wrote the manuscript.

### Conflict of interest statement

The authors declare that the research was conducted in the absence of any commercial or financial relationships that could be construed as a potential conflict of interest.
